# The Pretreatment Albumin to Globulin Ratio Has Predictive Value for Long-Term Mortality in Nasopharyngeal Carcinoma

**DOI:** 10.1371/journal.pone.0094473

**Published:** 2014-04-09

**Authors:** Xiao-Jing Du, Ling-Long Tang, Yan-Ping Mao, Ying Sun, Mu-Sheng Zeng, Tie-Bang Kang, Wei-Hua Jia, Ai-Hua Lin, Jun Ma

**Affiliations:** 1 Department of Radiation Oncology, Cancer Center, Sun Yat-sen University, Guangzhou, People’s Republic of China; 2 State Key Laboratory of Oncology in Southern China, Cancer Center, Sun Yat-sen University, Guangzhou, People’s Republic of China; 3 Department of Medical Statistics and Epidemiology, School of Public Health, Sun Yat-sen University, Guangzhou, People’s Republic of China; The University of Hong Kong, China

## Abstract

**Background:**

Low serum albumin is predictive of poor survival in nasopharyngeal carcinoma (NPC). We evaluated the ability of the pretreatment albumin/globulin ratio (AGR) to predict long-term mortality in patients with NPC.

**Methods:**

This retrospective study examined an unselected cohort of 694 patients with NPC who had documented pretreatment total serum protein and serum albumin levels (ALB). AGR was calculated as [AGR = ALB/(total serum protein - ALB)]. Survival analysis was used to evaluate the predictive value of AGR.

**Results:**

Multivariate analysis demonstrated that a low pretreatment serum AGR (<1.4) was an independent predictor of poor OS (*P*  = 0.029) and DMFS (*P*  = 0.033). A low AGR was significantly associated with advanced stage disease (*P*<0.001), high white blood cell count (*P*  = 0.033), high neutrophil count (*P*  = 0.047), high total serum protein (*P*<0.001) and low ALB (*P*<0.001).

**Conclusion:**

The pretreatment AGR may represent a simple, potentially useful predictive biomarker for evaluating the long-term prognosis of patients with undifferentiated NPC.

## Introduction

Nasopharyngeal carcinoma (NPC) is a common malignant disease in Southeast Asia, with an annual incidence of 30–80 per 10,000 [1]. Approximately 80% of patients present with advanced disease at first diagnosis as a result of its silent, deep-seated location and non-specific symptoms [2]. As it is radiosensitive, radiotherapy (RT) has long been the mainstay treatment for NPC. In recent years, developments in diagnostic methods, radiotherapeutic techniques and chemotherapy regimens have provided significant survival benefits in locally advanced NPC [3], [4]. However more than 20% of patients with advanced disease develop distant metastasis after treatment, making accurate prognostic evaluation at diagnosis extremely important for optimizing therapy [5], [6]. Current methods for assessing the prognosis of patients with NPC are mainly based on tumor-related factors. The TNM staging system, comprised of the T, N and M classifications, is the most commonly used method for determining the clinical treatment strategy and predicting treatment outcome. Additionally, a number of molecular biomarkers, such as the plasma Epstein–Barr viral DNA load, are established prognostic factors for overall survival and recurrence [7]. Nevertheless, it is becoming increasingly apparent that the prognostic value of these tumor-related factors in terms of disease progression is inadequate. A significant heterogeneity of treatment outcomes is observed for existing prediction models in NPC [8]. Thus, the discovery of biological markers, which can predict the risk of metastasis and mortality to assist with clinical decision-making, is still a major topic of translational research in NPC.

Malnutrition and cancer-related inflammation have been suggested to be crucial host-related factors that may negatively affect the outcome of cancer treatment, as they may promote tumor growth and metastasis by damaging the immune system and alter tumor cell biology in the tumor microenvironment [9], [10]. Albumin (ALB) is an important serum protein that reflects the patients’ nutritional status. Prior studies demonstrated that low serum ALB is an independent predictor of poor survival in several types of cancer including gastrointestinal cancer, lung cancer, ovarian cancer and breast cancer, as well as NPC [11], [12]. Globulins are the other major constituent of total serum proteins, and function as a carrier of sex hormones and play a major role in immunity and inflammation. In contrast to the considerable amount of research on ALB and other inflammation-related factors [12]–[18], the impact of the albumin/globulin ratio (AGR) on metastasis and mortality in NPC has not yet been addressed.

## Materials and Methods

### Patients

This was a retrospective study of an unselected cohort of all newly diagnosed patients with histologically proven, non-disseminated NPC who were hospitalized and treated at Sun Yat-sen University Cancer Center (Guangzhou, China) between January 2003 and December 2006. In all, 719 cases were evaluated, of whom 25 (3.5%) were subsequently eliminated, including 18 (2.5%) patients with incomplete laboratory data, 5 (0.7%) who were unable to complete the prescribed treatment, and 2 (0.3%) with other malignancies in addition to NPC. Thus, 694 patients were included in the analysis. The study was approved by the Institutional Review Board of Sun Yat-sen University Cancer Center. As this was a retrospective analysis of routine data, we requested and were granted a waiver of individual informed consent from the ethics committee. Patient records/information was anonymized and de-identified prior to analysis.

### Treatment

All patients underwent a pretreatment baseline evaluation including complete medical history, physical and neurological examinations, hematology and biochemistry profiles, MRI scan of the neck and nasopharynx, chest radiography and abdominal sonography. Treatment plans were determined according to standard protocols depending tumor stage and general health. All patients were treated with continuously definitive radiotherapy (RT) with daily fractions of 2.0 Gy and five fractions per week using a linear accelerator (6–8 MV). The radiation dose-ranges to the nasopharynx, lymph node-positive area and lymph node-negative area were 60–80, 60–70 and 50–60 Gy, respectively. Induction or adjuvant chemotherapy consisted of three cycles of cisplatin with 5-fluorouracil, or cisplatin with taxanes every 3 weeks. Concurrent chemotherapy consisted of cisplatin every 3 weeks or cisplatin weekly. In total, 479/694 (69.0%) patients received chemotherapy. Most of them (376/479, 78.5%) had advanced stage disease (classified as T3–T4 and/or N2–N3). When possible, salvage treatments, including afterloading, surgery and chemotherapy, were provided in the event of documented relapse or persistent disease.

### Data Collection

Clinicopathological characteristics were recorded at baseline before treatment. All patients enrolled in this study had serum chemistry analysis and complete blood counts within 14 days of any therapeutic intervention (with the exception of the diagnostic biopsies of the primary tumor or cervical lymph nodes).

Blood counts were performed using a Sysmex XE-5000 automated hematology analyzer (Sys- mex, Kobe, Japan). Serum ALB and total serum protein were determined using an automated immunoturbidmetric analyzer (7600-020; Hitachi High-Technologies, Tokyo, Japan). AGR was calculated using the equation AGR = ALB/(total serum protein – ALB). The cut-off value for ALB was 43 g/L, in accordance with the findings of Li et al. [12]. No patients had any coexistent hematologic disorders, known active infections or malnutrition before treatment; the ALB was higher than 35 g/L before treatment in all patients.

### Follow-up

After completion of therapy, patients were examined every 3 months during the first 2 years, and every 5 months thereafter for up to 6 years or until death. The end of follow-up was February 28^th^, 2013. The median follow-up time was 88 months (range, 5–123 months). No patients were lost to follow-up. The following end points (time to the first defining event) were assessed: distant metastasis-free survival (DMFS), overall survival (OS), disease-free survival (DFS) and local relapse-free survival (LRFS).

### Statistical Analysis

Statistical Package for the Social Sciences, version 20.0 (SPSS, Chicago, IL, USA) was used for all statistical analyses. The optimal cut-off value for classifying the pretreatment AGR as high or low for subsequent analysis was determined using the method of Igarashi et al. [19]. To do this, we stratified the patients using cut-off values from 1.0 to 2.4 in steps of 0.1, and then used the log-rank test to calculate DMFS curves. We selected the cut-off value for which the DMFS curves differed the most between the low and high AGR groups. This cut-off allowed us to treat serum AGR as a binary variable.

The distributions of continuous and categorical variables are presented as means ± standard deviations and frequencies and percentages, respectively. The Chi-square test was used for categorical variables and the Mann-Whitney U-test for continuous variables. Survival curves were made by the Kaplan–Meier method and compared by the log-rank test. Multivariate analysis using a Cox proportional hazards model was used to test independent significance by backward elimination of insignificant explanatory variables. Two-tailed *P*-values <0.05 were considered significant.

## Results

### Clinicopathological Features and Treatment Outcomes

The clinicopathological characteristics of the 694 patients, of whom 517 (74.5%) were male and 177 (25.5%) were female, are presented in Table 1. The median age at diagnosis was 44 years (range, 13–78). Based on the World Health Organization (WHO) criteria, 99.3% of patients had type II or III disease and 0.7% had type I disease. All patients were staged according to the 7th edition of the International Union against Cancer/American Joint Committee on Cancer (UICC/AJCC) staging system for NPC [20].

**Table 1:** Characteristics of the 694 patients with NPC.

Variable	No. (%)	Low AGR group (AGR <1.4)	High AGR group (AGR ≥1.4)	*P*-value
**Age, years**				0.073
<50	473 (68.2)	144 (30.4)	329 (69.6)	
≥50	221 (31.8)	82 (37.1)	139 (62.9)	
**Sex**				0.120
Male	517 (74.5)	160 (30.9)	357 (69.1)	
Female	177 (25.5)	66 (37.3)	111 (62.7)	
**T classification**				<0.001
T1-2	313 (45.1)	77 (24.6)	236 (75.4)	
T3-4	381 (54.9)	149 (39.1)	232 (60.9)	
**N classification**				0.077
N0	196 (28.2)	54 (27.6)	142 (72.4)	
N1-3	498 (71.8)	172 (34.5)	326 (65.5)	
**Clinical stage**				<0.001
I+II	235 (33.9)	54 (23.0)	181 (77.0)	
III+IV	459 (66.1)	172 (37.5)	287 (62.5)	
**Chemotherapy**				0.160
No	215 (31.0)	62 (28.8)	153 (71.2)	
Yes	479 (69.0)	164 (34.2)	315 (65.8)	
**Body mass index, kg/m^2^**		22.6±3.6	22.8±3.1	0.349
**Total protein, g/L**		78.2±5.7	74.5±5.6	<0.001
**ALB, g/L**		43.3±3.3	46.3±3.3	<0.001
**White blood cell count, k/cc**		7.4±2.2	7.1±2.3	0.033
**Neutrophil, k/cc**		4.7±2.0	4.4±2.0	0.047
**Lymphocyte, k/cc**		1.9±0.8	1.9±0.7	0.982

Abbreviations: AGR, albumin/globulin ratio; ALB, serum albumin.

During follow-up, 185/694 patients (26.7%) experienced tumor progression after treatment, including 70 (10.1%) who developed local or regional recurrence and 130 (18.7%) who developed distant metastasis. By the end of follow-up, 164/694 patients (23.6%) had died, including 150 (21.6%) who died of NPC and 14 (2.0%) who died of other causes. The 5-year OS, DFS, DMFS and LRFS rates were 82.6%, 76.2%, 83.6% and 89.9%, respectively.

### Identification of the Optimal Cut-off Value for Defining AGR as High or Low

The differences in the DMFS rates obtained when the patients were stratified into high or low AGR groups using various cut-off values are shown in Table 2. The largest difference was obtained using a cut-off of 1.4; this cut-off was used to define the low and high AGR groups in subsequent analyses.

**Table 2:** Differences in the distant metastasis-free survival rates for patients with NPC dichotomized using different cut-off values for the pretreatment albumin/globulin ratio.

		DMFS	
Cut-off, g/L	No. of patients, low/high	Chi-square	*P*-value
1.0	6/688	4.387	0.036
1.1	25/669	7.460	0.006
1.2	60/634	5.485	0.019
1.3	138/556	12.148	<0.001
1.4	226/468	12.579	<0.001
1.5	341/353	10.154	0.001
1.6	437/257	9.449	0.002
1.7	528/166	10.003	0.002
1.8	587/107	7.977	0.005
1.9	628/66	8.930	0.003
2.0	661/33	7.147	0.008
2.1	672/22	4.838	0.028
2.2	683/11	2.291	0.130
2.3	685/9	1.840	0.175
2.4	688/6	1.161	0.281

### Association of AGR with the Clinicopathological Features of NPC

The mean AGR in our patient population was 1.53, with a SD of 0.29. Based on the optimal cut-off value (<1.4), 226/694 patients (32.6%) had a low AGR level. The distribution of the AGR values differed significantly when the patients were stratified by T classification and clinical stage (Table 1). Significantly more patients classified as T3-4 were in the low AGR group than patients classified as T1-2 (*P*<0.001), and 54/235 (23.0%) and 172/459 (37.5%) of the patients with stage I+II and stage III+IV disease had a low AGR (*P*<0.001). Additionally, the patients in the low AGR group had a significantly higher white blood cell count (*P*  = 0.033), higher neutrophil count (*P*  = 0.047), higher total serum protein (*P*<0.001) and lower ALB (*P*<0.001) than the patients in the high AGR group.

### Predictive Value of AGR

**Figure 1:**
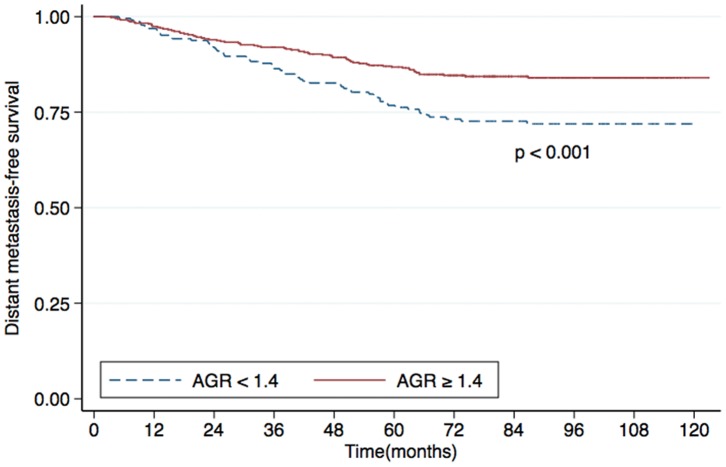
Distant metastasis-free survival (DMFS) rates for patients with nasopharyngeal carcinoma (NPC) with a pretreatment serum albumin/globulin ratio (AGR) <1.4 (dashed line) or ≥1.4 (solid line).

**Figure 2:**
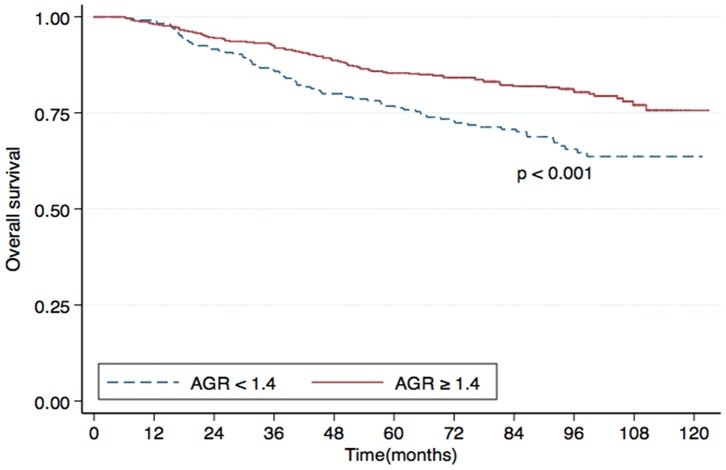
Overall survival (OS) rates for patients with nasopharyngeal carcinoma (NPC) with a pretreatment serum albumin/globulin ratio (AGR) <1.4 (dashed line) or ≥1.4 (solid line).

**Figure 3:**
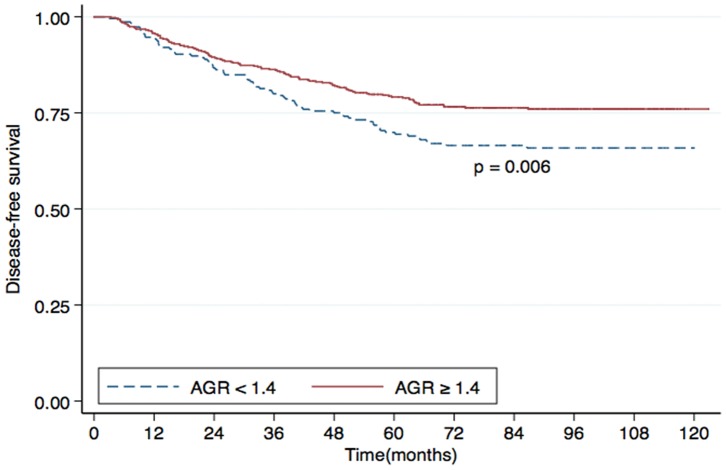
Disease-free survival (DFS) rates for patients with nasopharyngeal carcinoma (NPC) with a pretreatment serum albumin/globulin ratio (AGR) <1.4 (dashed line) or ≥1.4 (solid line).

**Figure 4:**
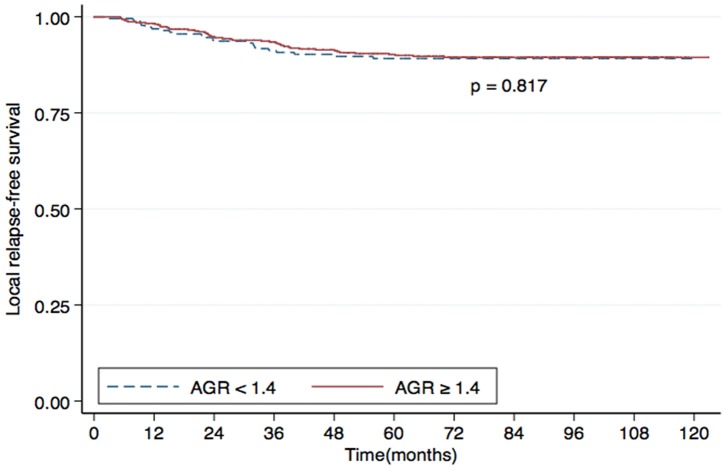
Local relapse-free survival (LRFS) rates for patients with nasopharyngeal carcinoma (NPC) with a pretreatment serum albumin/globulin ratio (AGR) <1.4 (dashed line) or ≥1.4 (solid line).

Univariate analysis identified the AGR as a statistically significant predictive factor for DMFS (*P*<0.001; Figure 1), OS (*P*<0.001; Figure 2) and DFS (*P*  = 0.006; Figure 3). However, the AGR had no predictive value for LRFS (*P*  = 0.817; Figure 4). Multivariate analysis for DMFS, OS and DFS was performed to adjust for various prognostic factors. The following parameters were included in the Cox proportional hazards model: age (≥50 vs. <50 years), gender (male vs. female), T classification (T3-4 vs. T1-2), N classification (N1-3 vs. N0), chemotherapy (yes vs. no), pretreatment ALB (<43 g/L vs. ≥43 g/L) and pretreatment AGR (<1.4 vs. ≥1.4). Total serum protein was excluded, as it was used in calculation of the AGR. Consistent with the univariate analysis, the AGR was an independent predictive factor for DMFS and OS (*P*  = 0.033 and 0.029, respectively). However, the AGR was not a significant predictive factor for DFS in multivariate analysis. T classification and ALB were also identified as independent predictive factors for DMFS, OS and DFS in multivariate analysis. Furthermore, N classification was an independent predictive factor for DFS and DMFS, and chemotherapy was significant for OS. In contrast, patient age and gender were not independent predictive factors for any survival endpoint (Table 3).

**Table 3:** Multivariate analysis of prognostic factors in 694 patients with nasopharyngeal carcinoma.

	DMFS		OS		DFS	
Variable	*P*-value	HR (95% CI)	*P*-value	HR (95% CI)	*P*-value	HR (95% CI)
**Age** (≥50 vs. <50 years)					0.082	1.307 (0.967–1.766)
**Sex** (male vs. female)						
**T classification** (T3-4 vs. T1-2)	0.021	1.548 (1.067–2.246)	0.046	1.416 (1.006–1.995)	0.011	1.485 (1.095–2.013)
**N classification** (N1-3 vs. N0)	0.011	1.786 (1.144–2.788)			0.008	1.624 (1.137–2.319)
**Chemotherapy** (yes vs. no)			0.051	1.468 (0.999–2.158)		
**ALB** (<43 g/L vs. ≥43 g/L)	0.043	1.486 (1.012–2.183)	0.002	1.716 (1.227–2.401)	0.001	1.684 (1.235–2.297)
**AGR** (≥1.4 vs. <1.4)	0.033	1.489 (1.033–2.145)	0.029	1.439 (1.038–1.994)		

Abbreviations: AGR, albumin/globulin ratio; ALB, serum albumin; OS, overall survival; DFS, disease-free survival; DMFS, distant metastasis-free survival; 95% CI: 95% confidence interval; HR: hazard ratio.

## Discussion

This study demonstrates that the pretreatment AGR is a significant predictor of long-term mortality in NPC. Although a low AGR was associated with a higher clinical stage, the predictive value of the AGR remained significant after adjustment for T classification, N classification and other clinical characteristics in multivariate analysis. Patients with a low pretreatment AGR had poorer DMFS and OS rates than those in the high AGR group.

In this study, the AGR was calculated as AGR = ALB/(total serum protein – ALB). As total serum protein includes many other types of inflammatory proteins in addition to globulins (*i.e.,* C-reactive protein [CRP], interleukins, leukotrienes, among others), the AGR is the ratio of ALB to the non-albumin proteins (*i.e.,* globulins and other inflammatory proteins). Previous studies showed that a low AGR was predictive for poor survival in breast cancer [21] and colorectal cancer [22]. To the best of our knowledge, this is the first study to specifically focus on the predictive value of the AGR in NPC.

ALB is the most abundant serum protein. In adults, the normal ALB range is 35–50 g/L; levels <35 g/L are termed hypoalbuminemia [11]. ALB is most commonly used to assess nutritional status, and is also a useful factor for predicting the prognosis of patients with cancer. Research conducted over the last decade or so has demonstrated associations between a low ALB and an increased severity of disease, a high risk of disease progression and poor survival in several types of cancer [11], [23]. One study reported that although most patients with NPC had ALB values in the normal range at diagnosis, a pretreatment ALB <43 g/L was predictive of poor prognosis [12]. Several mechanisms have been proposed to explain the anticancer effects of ALB, including its ability to stabilize cell growth and DNA replication, buffer a variety of biochemical changes, and maintain calcium and sex hormone homeostasis to protect against sex hormone-induced cancers, as well as its antioxidant effects against carcinogens such as nitrosamine and aflatoxin [24]. In vitro, the proliferation or growth of cancer cell lines (i.e., human breast cancer cell lines) is inhibited by high concentrations of albumin [24]–[26]. In addition, malnutrition, which is very common among patients with cancer and is reflected by low ALB, can weaken a number of human defense mechanisms including anatomic barriers, cellular and humoral immunity, and phagocyte function, thus increasing susceptibility to infection and further compromising the response to treatment [27].

On the other hand, malnutrition and inflammation can suppress the synthesis of albumin [11]. As a part the systemic inflammatory response against the tumor, high levels of proinflammatory cytokines and growth factors are released by the tumor or surrounding cells, which in turn alters metabolic homeostasis in the tumor microenvironment [10]. For example, interleukin-6 stimulates the production of acute-phase reaction proteins in the liver and modulates the production of albumin by hepatocytes [11], [28], whereas tumor necrosis factor can inhibit transcription of the albumin gene and increase the permeability of the microvasculature leading to increased transcapillary passage of albumin [24], [29].

High levels of globulins arise due to elevated accumulation of acute-phase proteins and immunoglobulins, as well as other serum proteins; these changes are reflective of an inflammatory state. It has been reported that approximately 25% of cancers are initiated or promoted by chronic inflammation associated with infection or inflammatory conditions of diverse origin. Additionally, other types of cancer that are not associated with inflammation are usually characterized by the presence of inflammatory cells and mediators [30]. There are several reasons why cancer-related inflammation is especially important in NPC. First, a consistent, massive leukocytic infiltrate is present in virtually all primary tumors, and is suspected to enhance the malignant growth of NPC cells [31]. One study reported a very low rate for the establishment of successful NPC xenografts when using fragments from primary tumors (about 1%) in contrast with approximately 50% for metastatic fragments, possibly reflecting the crucial role of the leukocyte infiltrate on tumor development, particularly for the primary tumor [32]. Additionally, Epstein–Barr viral infection is associated with NPC in the areas where NPC is endemic, and induces the consistent expression of viral immunogenic proteins leading to a potent immune response [7], [31]. Finally, high levels of numerous cytokines and other inflammation-related factors are consistently detected in the peripheral blood of patients with NPC [31].

In the present study, we observed significant associations between a low AGR and higher leukocyte and neutrophil counts, consistent with previous research in breast cancer [21]. It has been reported that a high peripheral neutrophil level, which was induced by inflammation-related cytokines (ie, interleukin-6 and tumor necrosis factor) or tumor-derived myeloid growth factors, may indicate systemic inflammation response or a tumor progression. It is because neutrophil can produce proangiogenic factors (ie, vascular endothelial growth factor) to assist tumor aggressiveness [14], [33].

The systemic inflammatory response is a non-specific response secondary to tumor hypoxia and necrosis or local tissue damage [34]. There is good evidence that a chronic systemic inflammatory response is clearly related to progressive nutritional decline, a poor response to treatment and poor prognosis in patients with cancer [10], [34]–[35]. The acute-phase protein CRP is mainly synthesized and released into the systemic circulation by hepatocytes, and is used as a non-specific marker of inflammation. The magnitude of the increase in CRP levels has been associated with poor survival in cancer, particularly in patients with advanced stage disease [17], [34]. A previous study demonstrated that a high CRP level (≥2.46 mg/L) was predictive of poor OS and DMFS in NPC [17], [18]. Assessment of the systemic inflammatory response was subsequently refined using a selective combination of hematological components to create inflammation-based prognostic factors [35]. A high neutrophil to lymphocyte ratio has been associated with poor survival in NPC [13]–[15], whereas a high lymphocyte to monocyte ratio has been reported to be a significant predictor of a favorable prognosis in NPC [16].

We believe that nutritional status and the systemic inflammatory response play a major role in the progression and metastasis of NPC. The AGR is a combination of these two predictors of adverse outcome, which may enhance its predictive value. We view the AGR in theory to be a superior predictive factor compared to other indicators of nutrition or inflammation, and suggest that the AGR should be assessed prior to treatment in patients with NPC. Nutritional assessment and support, as well as anti-inflammatory therapy, may be suitable treatment choices in NPC, particularly for patients with an AGR <1.4. Currently, there are several ongoing studies investigating the ability of anti-inflammatory therapy (*e.g*., aspirin and other non-steroidal anti-inflammatory drugs) to prevent and/or treat lung, esophageal, stomach, colon and bladder cancer [36], [37], and the value of anti-inflammatory therapy in NPC needs to be explored further.

There are some limitations to the current study. The AGR was calculated indirectly from total serum protein and ALB. Although CRP and other serum proteins are also important indicators of cancer-related inflammation, these factors were not routinely measured at our hospital prior to 2006; therefore, the long-term predictive value of these factors could not be assessed. In addition, the AGR was only assessed at a single time point before treatment. The changes in serum chemistry and complete blood counts over time and in response to treatment, and their relationship with survival are of considerable interest and will be the subject of future work.

Despite these limitations, this study is informative. Our findings identify a direct relationship between the pretreatment AGR and long-term mortality in NPC, and suggest that the AGR represents a clinical biomarker which could potentially be modulated to improve patient prognosis. As the AGR is routinely measured at low cost in clinical practice, it has potential as a simple, convenient predictive and stratification factor to assist with clinical decision-making in NPC.
